# Effects of straw return with potassium fertilizer on the stem lodging resistance, grain quality and yield of spring maize (*Zea mays* L.)

**DOI:** 10.1038/s41598-023-46569-z

**Published:** 2023-11-20

**Authors:** Jian Liu, Ya-fang Fan, Ji-ying Sun, Ju-lin Gao, Zhi-gang Wang, Xiao-fang Yu

**Affiliations:** 1 College of Agronomy, Inner Mongolia Agricultural University, No.275, XinJian East Street, Hohhot, 010019 China; 2 Plant Protection Institute, Inner Mongolia Academy of Agricultural & Animal Husbandry Sciences, Hohhot, 010031 China; 3 Vocational and Technical College, Inner Mongolia Agricultural University, Baotou, 014109 China

**Keywords:** Biochemistry, Ecology, Plant sciences, Ecology

## Abstract

This experiment aimed to study the effects of straw return combined with potassium fertilizer on stem lodging resistance, grain quality, and yield of spring maize. The objective was to provide a scientific basis for the rational utilization of Inner Mongolia spring maize straw and potassium fertilizer resources. The test material used was ‘Xianyu 335’, and the study was conducted in three ecological regions from east to west of Inner Mongolia (Tumochuan Plain Irrigation Area, Hetao Plain Irrigation Area, and Lingnan Warm Dry Zone). A split-plot design was employed, with the straw return method as the main plot and potassium fertilizer dosage as the secondary plot. We determined the stem resistance index, grain quality, and yield. The results showed that both straw return and potassium application improved stem lodging resistance, grain quality, and maize yield. Combining straw return with the reasonable application of potassium fertilizer enhanced the effectiveness of potassium fertilizer, increased lodging resistance, maize yield, and improved grain quality and yield stability. Under the straw return treatment, with potassium application compared to no potassium application, significant increases were observed in maize plant height, stem diameter, dry weight of stems, stem compressive strength, stem bending strength, grain protein content, yield, straw potassium accumulation content, and soil available potassium content. These increases were up to 30.79 cm, 2.63 mm, 15.40 g, 74.93 N/mm^2^, 99.65 N/mm^2^, 13.68%, 3142.43 kg/hm^2^, 57.97 kg/hm^2^, and 19.80 mg/kg, respectively. Therefore, the interaction of straw return and potassium fertilizer was found to be the most effective measure for maintaining high-yield and stress-resistant cultivation, improving grain quality, and optimizing the management of straw and potassium fertilizer resources. This approach is suitable for promotion and application in the spring maize growing areas of Inner Mongolia.

## Introduction

As the most popular grain crop in China, maize (*Zea mays* L.) plays a crucial role in ensuring a stable increase in national grain production and food security^1^. The goals of the maize growing industry in China have shifted from solely focusing on high yields to a more comprehensive approach that emphasizes optimizing the structure, improving quality, and controlling costs. Therefore, current agricultural strategies are adjusting cultivation methods, improving the quality of maize products, and promoting ecologically responsible and high-quality development in the planting industry based on ensuring a stable increase in grain production.

Maize lodging, which refers to the bending or breaking of maize plants, is a significant factor that limits maize yields. Previous reports from the past decade have shown that the lodging rate of maize is positively correlated with ear height^2,3^, and for every 1% increase in the lodging rate, the yield decreases by 108 kg/hm^2^. The mechanical properties associated with maize stem lodging resistance serve as critical indicators of the degree of lodging resistance in maize plants and are negatively correlated with field lodging rates. The phenotypic characteristics of stems influence these mechanical properties and determine the lodging resistance of plants^4–6^. Considering the evolving direction of grain production practices in China, achieving stable production and guaranteed income necessitates improvements in grain development, maize grain quality, and economic efficiency. Long-term fertilization impacts the quality of maize grain^7^. In addition, genetics, fertilization measures, and environmental conditions also influence grain quality^8^. Straw return provides abundant nutrients. Straw return can promote the synthesis of crude fat, protein and starch in maize grain, thereby improving grain quality^9^.

Potassium fertilizer plays a crucial role in enhancing the lodging resistance, quality, and yield of maize^10^.

However, China faces a challenge as it lacks potassium resources and heavily relies on imported fertilizers, leading to high costs and limited supply. The effectiveness of K^+^ in both soil and fertilizer is dependent on the soil conditions^11^. To address the increasing issue of potassium deficiency in the soil, it is necessary to supplement potassium in addition to using potassium fertilizer. Considering the current level of soil productivity, it becomes essential to explore the soil's inherent production potential to enhance soil nutrient content, improve soil structure and physicochemical properties, optimize the ecological environment of farmland, and maintain high crop yields. This approach aims to avoid resource wastage and environmental pollution^12–14^. The improper disposal of straw resources through direct burning, abandonment, or incineration not only causes significant environmental pollution but also leads to substantial resource wastage. Therefore, returning straw to the soil can optimize soil structure, physical properties, and chemical properties^15^. Straw return plays a vital role in enhancing soil structure, physical and chemical properties, boosting soil enzyme activity and soil nutrients, and ultimately increasing maize yield^16,17^. China is rich in straw resources. The average annual maize stover yield in China is 399.18 million t, and the potassium nutrient content of the returning maize stover in China is 4.79 million t K_2_O. The potassium fertilizer substitution potential of maize straw returned in the growing season in China is 24.4 kg/hm^2^ K_2_O^18^, and the release rate of potassium during this season is approximately 85%^19^. It is generally believed that using straw resources can alleviate soil potassium deficiency, enrich the soil potassium pool, and improve soil fertility. By using straw resources, farmers can meet the potassium requirements for maize production in China.

Previous studies have primarily focused on the addition of potassium fertilizer to increase maize yield through straw return^20,21^. However, there is limited research on the impact of straw return combined with potassium fertilizer on stem lodging resistance, grain quality, and yield of spring maize. In this study, we investigated the effects of straw return with potassium fertilizer on phenotypic traits, mechanical properties of stem lodging strength, grain quality, and yield of spring maize across three ecological regions in Inner Mongolia, spanning from east to west. Our findings contribute to the development of a cultivation model suitable for producing high-quality and high-yield maize agriculture. This study provides a basis for cultivating high-yield and stress-resistant spring maize in Inner Mongolia and elsewhere in China. At the same time, it also laid the foundation for developing the high-quality green planting industry.

## Materials and methods

### Site description

In 2020, we selected three ecological regions in Inner Mongolia as our test sites: Tumochuan Plain Irrigation Area, Hetao Plain Irrigation Area, and Lingnan Warm Dry Zone. Table 1 provides information on the longitude, latitude, sunshine duration from April to October, average temperature, and rainfall at each test location. Table 2 presents the soil type and baseline soil fertility of each test site.

**Table 1:** Latitude, longitude and climatic conditions of three ecological regions in Inner Mongolia.

Ecological region	Experimental sites	Latitude	Longitude	Sunshine duration (h)	Average temperature (℃)	Rainfall (mm)
Hetao plain irrigation area	Bayannur	41°11′ N	122°49′ E	1924.3	20.4	157.6
Tumochuan plain irrigation area	Baotou	40°32′ N	122°48′ E	1716.4	21.1	317.7
Lingnan warm dry zone	Xing’an	46°45′ N	122°47′ E	1330.5	22.3	392.5

**Table 2:** Soil type and soil basic fertility of three ecological regions in Inner Mongolia.

Ecological region	Soil type	Organic Matter (g/kg)	Total N (g/kg)	Available N (mg/kg)	Olsen P (mg/kg)	Available K (mg/kg)	pH
Hetao plain irrigation area	Irrigated soil	26	0.4	87.5	6.6	140.3	7.9
Tumochuan plain irrigation area	Silty loam	26.7	0.5	92.4	8.8	118	7.6
Lingnan warm dry zone	Black soil	29.1	1.3	100.6	13.7	112.8	7.8

The experimental research and field studies on plants (either cultivated or wild), including the collection of plant material, comply with relevant institutional, national, and international guidelines and legislation. The field study was carried out on official land belonging to the Key Laboratory of Crop Cultivation and Genetic Improvement of the Inner Mongolia Autonomous Region. Permission was given after the research application passed verification, and the studies complied with local and national regulations. During the field study, our test did not involve endangered or protected species. No specific permissions were needed for conducting the field study because it was not carried out in a protected area.

### Experimental design

In this study, ‘Xianyu 335’ was used as the test material in a split plot design, where the straw return method was the main plot and the potassium fertilizer dosage was the subplot. ‘Xianyu 335’ is a maize hybrid bred by American Vanguard Corporation. The female parent of Xianyu 335 is PH6WC, and the male parent is PH4CV. Both the female and male parents are bred by American Vanguard Corporation. The study included four treatments: (ST+6K), straw return (ST+0K), potassium fertilizer (NST+6K), no straw return and no potassium fertilizer (NST+0K). For the plots with straw return, all the ground portions of straw were crushed and returned to the soil in the previous autumn. For the plots without straw return, the straw was removed from the treatment areas after harvesting. Each experimental treatment was represented by a plot measuring 30 m in length and 5 m in width, with a total area of 150 m^2^ Five repetitions were conducted for each plot, and the planting density was 82,500 plants/hm^2^. Maize straw is rich in nutrients such as nitrogen, phosphorus, and potassium. The average nutrient content of maize straw is 9.2 g/kg of nitrogen, 3.5 g/kg of phosphorus, and 14.2 g/kg of potassium.

As basic fertilizer, potassium was applied once before sowing in the form of 90 kg/hm^2^ potassium sulfate (K_2_O 50%) and 228 kg/hm^2^ diammonium phosphate (P_2_O_5_ 46%). In the treatments without potassium, only 228 kg/hm^2^ diammonium phosphate (P_2_O_5_ 46%) was applied once as basic fertilizer before sowing. The top dressing of each treatment was 652 kg/hm^2^ (N 46%), which was applied in the jointing stage and the bell stage at a ratio of 3:7. Other management practices followed typical field production practices^22^.

### Measurements

Soil basic fertility. Prior to sowing, soil samples were collected from each treatment at a depth of 0–20 cm and passed through a 0.15–0.25 mm soil sieve for analysis. The analysis included determining soil organic matter, soil total nitrogen, soil available nitrogen, soil available phosphorus, and soil available potassium. We referred to the Soil Agrochemical Analysis to study the specific measurement methods for the above indicators^23^.

Stem indicators of maize. During the silking period, the following maize stem indicators were measured. Plant height, ear height, and ear stem length were measured using a steel ruler. The diameter of the maize stem at the third stem node near the base was determined using a caliper. Stem fresh weight and stem dry weight were measured using an electronic balance^24^. The Brix of the maize stems was measured using a portable digital sugar meter (PAL-1, Japan ATAGO, accuracy =  ± 0.2%)^25^. Stem puncture strength, stem compressive strength, and stem bending strength were determined at the third stem node near the base using a plant stem strength instrument (YYD-1, Tuopu Yunnong, Zhejiang, accuracy =  ± 0.5% F.S.)^26^.

Maize grain quality^27^. At physiological maturity, we measured the starch content of maize grains, crude fat content of maize grains, protein content of maize grains, and water content of maize grains with a FOSS near-infrared grain quality analyser (Infratee TM 1241, FOSS, Denmark).

Yield and yield components^28,29^. At physiological maturity, we harvested four rows (20 m^2^) of maize in the middle of each plot. Ten plants with uniform ear growth were selected to measure the maize grain number per ear, 1000-grain weight, and water content, and the maize yield was calculated. The water content was measured using an LDS-1G moisture content detector.

### Statistics analysis

We used the Data SPSS window version 17 (SPSS Inc., Chicago, USA) to finish the statistical analysis. Under straw return treatments, potassium fertilizer treatments, and ecological regions, we examined the stem lodging resistance, grain quality, and yield of spring maize using GLM based on the model for a split-plot design^30^. Straw return treatments, potassium fertilizer treatments, and ecological regions were the independent variables, and the stem lodging resistance, grain quality, and yield of spring maize were dependent variables in this test. To determine the impact of independent variables on dependent variables, we used three-way analysis of variance to test statistically significant variance, and we used the least significant difference (LSD) test with α = 0.05 to make multiple comparisons^31^. We used SigmaPlot 12.5 to make histograms. Different letters on the histograms indicate significant differences at the P < 0.05 level.

## Results

### Effects of straw return combined with potassium fertilizer on the morphological indexes of spring maize stems

The analysis of variance (ANOVA) results revealed that the straw return method, potassium fertilizer dosage, and ecological region had a significant impact on plant height, ear height, stem diameter, and ear stem length (Table 3). There were significant interactions between the straw return method and potassium fertilizer dosage in relation to the above indicators. Additionally, the interactions between the straw return method and ecological region had a significant influence on plant height and ear stem length.

**Table 3:** ANOVA results for maize stem morphological indicators under different straw return methods and potassium fertilizer treatments.

Source of variation	DF	Plant height	Ear height	Stem diameter	Stem length
S	1	107.26***	29.38***	21.88***	509.17***
K	1	426.34***	193.91***	49.33***	1121.14***
E	2	30.94***	28.10***	45.69***	45.85***
S × K	1	37.67***	18.13***	6.84**	52.25***
S × E	2	4.70**	1.47	0.18	8.69***
K × E	2	1.48	0.52	0.38	1.64
S × K × E	2	1.39	0.75	0.05	0.16

Note: S, K, and E represent straw return treatments, potassium fertilizer treatments, and ecological regions, respectively. Values in the table are F-values with corresponding degrees of freedom for each of the simple effects and two and three way interactions with *, **, *** indicating significant effects at respectively 5%, 1%, 0.1%

As shown in Table 4, the maize plant height, ear height, ear stem diameter, and ear stem length varied among the treatment groups. In the Tumochuan Plain Irrigation Area, the straw return treatment resulted in significant increases in maize plant height, ear height, stem diameter, and ear stem length. Specifically, under the treatment with straw return, the maize plant height, ear height, stem diameter, and ear stem length increased by 23.69 cm, 9.88 cm, 2.17 mm, and 1.45 cm, respectively, with potassium application compared to no potassium application. These increases corresponded to growth improvements of 7.74%, 8.35%, 8.54%, and 11.20%, respectively. Similarly, under the treatment without straw return, the addition of potassium led to increases of 14.93 cm, 6.20 cm, 1.03 mm, and 0.88 cm in maize plant height, ear height, stem diameter, and ear stem length, respectively, with potassium application compared to no potassium application. These increases represented growth improvements of 4.92%, 5.24%, 4.12%, and 7.17%, respectively. Notably, straw return further enhanced the effectiveness of potassium application, resulting in additional growth improvements of 2.82%, 3.11%, 4.42%, and 4.03% for maize plant height, ear height, stem diameter, and ear stem length, respectively.

**Table 4:** Effects of the interaction of the straw return method and potassium fertilizer dosage on the morphological indicators of maize stems in different ecological regions.

Ecological region	Straw return method	Potassium fertilizer dosage	Plant height (cm)	Ear height (cm)	Stem diameter (mm)	Stem length (cm)
Tumochuan plain irrigation area	ST	6 K	329.72 ± 3.82a	128.17 ± 2.33a	27.58 ± 1.15a	14.40 ± 0.11a
		0 K	306.03 ± 3.87c	118.29 ± 2.34c	25.41 ± 1.16c	12.95 ± 0.13c
	NST	6 K	318.10 ± 4.69b	124.43 ± 2.43b	26.01 ± 0.85b	13.16 ± 0.10b
		0 K	303.17 ± 3.73d	118.23 ± 3.01c	24.98 ± 0.91d	12.28 ± 0.24d
Hetao plain irrigation area	ST	6 K	340.63 ± 2.28a	133.59 ± 2.71a	29.75 ± 0.84a	14.63 ± 0.13a
		0 K	309.84 ± 2.63c	120.73 ± 2.13c	27.12 ± 0.96c	13.06 ± 0.14c
	NST	6 K	318.14 ± 4.35b	125.61 ± 2.09b	27.80 ± 0.86b	13.77 ± 0.18b
		0 K	303.81 ± 4.68d	119.91 ± 1.76c	26.38 ± 1.11d	12.68 ± 0.19d
Lingnan warm dry zone	ST	6 K	325.24 ± 2.57a	126.27 ± 3.02a	26.61 ± 1.05a	14.35 ± 0.11a
		0 K	301.11 ± 3.81c	116.08 ± 2.75c	24.32 ± 0.95c	12.84 ± 0.13c
	NST	6 K	310.95 ± 4.11b	120.47 ± 1.29b	24.77 ± 0.74b	13.13 ± 0.06b
		0 K	297.62 ± 3.98d	114.85 ± 1.44c	23.96 ± 0.70d	12.18 ± 0.11d

Note: ST represents the straw return treatment. NST represents the non-straw-return treatment. 6 K and 0 K represent the potassium fertilizer dosages.Values are means ± standard deviation; n = 5. Values followed by different letters in a column are significant between treatments using a LSD test (P < 0.05).

In the Hetao Plain Irrigation Area, the straw return treatment resulted in significant increases in maize plant height, ear height, stem diameter, and ear stem length. Specifically, under the treatment with straw return, the maize plant height, ear height, stem diameter, and ear stem length increased by 30.79 cm, 12.86 cm, 2.63 mm, and 1.57 cm, respectively, with potassium application compared to no potassium application. These increases corresponded to a percentage increase of 9.94%, 10.65%, 9.70%, and 12.02%, respectively. Similarly, under the treatment without straw return, the maize plant height, ear height, stem diameter, and ear stem length increased by 14.33 cm, 5.70 cm, 1.42 mm, and 1.09 cm, respectively, with potassium application compared to no potassium application. These increases represented a percentage increase of 4.72%, 4.75%, 5.38%, and 8.60%, respectively. The results indicated that straw return enhanced the effectiveness of potassium application on maize plant height, ear height, stem diameter, and ear stem length by 5.22%, 5.90%, 4.31%, and 3.43%, respectively.

In the Lingnan Warm Dry Zone, the straw return treatment resulted in significant increases in maize plant height, ear height, stem diameter, and ear stem length. Specifically, under the treatment with straw return, the maize plant height, ear height, stem diameter, and ear stem length increased by 24.13 cm, 10.19 cm, 2.29 mm, and 1.51 cm, with potassium application compared to no potassium application. These increases corresponded to a percentage increase of 8.01%, 8.78%, 9.42%, and 11.76%, respectively. Similarly, under the treatment without straw return, the maize plant height, ear height, stem diameter, and ear stem length increased by 13.33 cm, 5.62 cm, 0.81 mm, and 0.95 cm, equivalent to increases of 4.48%, 4.89%, 3.38%, and 7.80%, respectively, with potassium application compared to no potassium application. Straw return enhanced the effectiveness of potassium application on maize plant height, ear height, stem diameter, and ear stem length by 3.53%, 3.89%, 6.04%, and 3.96%, respectively.

### Effects of straw return with potassium fertilizer on the phenotypic traits of spring maize stems

The results of the analysis of variance (ANOVA) indicated that the straw return method, potassium fertilizer dosage, and ecological region had a significant impact on the stem fresh weight, stem dry weight, and stem brix (Table 5). There were significant interactions between the straw return method and potassium fertilizer dosage, which affected the above indicators. Both the straw return method and potassium fertilizer dosage had a significant influence on the water content of the stems. The interactions between the straw return method and ecological region also had a significant impact on the fresh weight and dry weight of the stems. The interactions between potassium fertilizer dosage and ecological region significantly affected the stem fresh weight. The interactions between the straw return method, potassium fertilizer dosage, and ecological region collectively had a significant effect on the stem fresh weight.

**Table 5:** ANOVA results for maize stem phenotypic traits under different straw return methods and potassium fertilizer treatments.

Source of variation	DF	Fresh weight	Dry weight	Water content	Brix
S	1	913.21***	82.14***	26.60***	640.87***
K	1	3963.88***	471.06***	83.46***	1194.27***
E	2	119.74***	46.06***	2.65	17.58***
S × K	1	315.18***	29.71***	3.46	53.18***
S × E	2	28.11***	3.28*	0.49	0.21
K × E	2	11.81***	1.77	2.98	1.14
S × K × E	2	4.31*	0.38	0.03	0.00

Note: S, K, and E represent straw return treatments, potassium fertilizer treatments, and ecological regions, respectively. Values in the table are F-values with corresponding degrees of freedom for each of the simple effects and two and three way interactions with *, **, *** indicating significant effects at respectively 5%, 1%, 0.1%

According to Table 6, there were variations in the maize fresh weight, dry weight, water content, and brix of stems among the different treatments. In the Tumochuan Plain Irrigation Area, the straw return treatment resulted in significant increases in maize fresh weight, dry weight, water content, and brix of stems. Specifically, under the treatment with straw return, the maize fresh weight, dry weight, water content, and brix of stems increased by 83.72 g, 15.40 g, 2.67%, and 1.64%, respectively, with potassium application compared to no potassium application. These increases corresponded to growth improvements of 44.19%, 30.00%, 3.66%, and 15.28%. Similarly, under the treatment without straw return, the addition of potassium led to increases of 50.65 g, 10.49 g, 1.53%, and 1.10% in maize fresh weight, dry weight, water content, and brix of stems, respectively, with potassium application compared to no potassium application. These increases represented growth improvements of 28.21%, 21.09%, 2.12%, and 10.95%, respectively. Notably, straw return further enhanced the effectiveness of potassium application, resulting in additional growth improvements of 15.98%, 8.91%, 1.55%, and 4.34% for maize fresh weight, dry weight, water content, and brix of stems, respectively.

**Table 6:** Effects of the interaction of the straw return method and potassium fertilizer dosage on the phenotypic traits of maize stems in different ecological regions.

Ecological region	Straw return method	Potassium fertilizer dosage	Fresh weight (g)	Dry weight (g)	Water content (%)	Brix (%)
Tumochuan plain irrigation area	ST	6 K	273.16 ± 3.01a	66.73 ± 2.77a	75.57 ± 0.99a	12.37 ± 0.20a
		0 K	189.44 ± 2.82c	51.33 ± 1.58c	72.90 ± 0.88c	10.73 ± 0.11c
	NST	6 K	230.17 ± 3.67b	60.23 ± 2.18b	73.82 ± 1.31b	11.15 ± 0.26b
		0 K	179.52 ± 1.59d	49.74 ± 1.86d	72.29 ± 0.98d	10.05 ± 0.12d
Hetao plain irrigation area	ST	6 K	294.74 ± 4.82a	71.17 ± 3.27a	75.85 ± 1.07a	12.44 ± 0.12a
		0 K	202.76 ± 4.89c	56.98 ± 1.65c	71.89 ± 0.78c	10.93 ± 0.16c
	NST	6 K	230.20 ± 7.43b	60.89 ± 2.11b	73.53 ± 1.07b	11.25 ± 0.06b
		0 K	183.29 ± 3.06d	53.78 ± 1.70d	70.64 ± 1.28d	10.29 ± 0.11d
Lingnan warm dry zone	ST	6 K	257.51 ± 3.52a	63.21 ± 2.11a	75.44 ± 1.10a	12.22 ± 0.13a
		0 K	183.85 ± 3.76c	49.45 ± 1.64c	73.09 ± 1.12c	10.67 ± 0.17c
	NST	6 K	216.83 ± 3.52b	56.82 ± 1.15b	73.79 ± 0.95b	10.97 ± 0.09b
		0 K	174.73 ± 2.71d	48.48 ± 1.83d	72.25 ± 1.02d	9.97 ± 0.07d

Note: ST represents the straw return treatment. NST represents the non-straw-return treatment. 6 K and 0 K represent the potassium fertilizer dosages.Values are means ± standard deviation; n = 5. Values followed by different letters in a column are significant between treatments using a LSD test (P < 0.05).

In the Hetao Plain Irrigation Area, the straw return treatment resulted in significant increases in maize fresh weight, dry weight, water content, and brix of stems. Specifically, under the treatment with straw return, the maize fresh weight, dry weight, water content, and brix of stems increased by 91.98 g, 14.19 g, 3.96%, and 1.51%, respectively, with potassium application compared to no potassium application. These increases corresponded to a percentage increase of 45.36%, 24.90%, 5.51%, and 13.82%, respectively. Similarly, under the treatment without straw return, the maize fresh weight, dry weight, water content, and brix of stems increased by 46.91 g, 7.11 g, 2.89%, and 0.96%, respectively, with potassium application compared to no potassium application. These increases represented a percentage increase of 25.59%, 13.22%, 4.09%, and 9.33%, respectively. Straw return enhanced the effectiveness of potassium application on the maize fresh weight, dry weight, water content, and brix of stems by 19.77%, 11.68%, 1.42%, and 4.49%, respectively.

In the Lingnan Warm Dry Zone, the straw return treatment resulted in significant increases in maize fresh weight, dry weight, water content, and brix of stems. Specifically, under the treatment with straw return, the maize fresh weight, dry weight, water content, and brix of stems increased by 73.66 g, 13.76 g, 2.35%, and 1.55%, respectively, with potassium application compared to no potassium application. These increases corresponded to a percentage increase of 40.07%, 27.83%, 3.22%, and 14.53%, respectively. Similarly, under the treatment without straw return, the maize fresh weight, dry weight, water content, and brix of stems increased by 42.10 g, 8.34 g, 1.54%, and 1.00%, respectively, with potassium application compared to no potassium application. It represented the increases of 24.09%, 17.20%, 2.13%, and 10.03%, respectively. Straw return enhanced the effectiveness of potassium application on the maize fresh weight, dry weight, water content, and brix of stems by 15.97%, 10.62%, 1.08%, and 4.50%, respectively.

### Effects of the straw return method and potassium fertilizer dosage on the lodging resistance mechanical properties of spring maize stems

The results of the analysis of variance (ANOVA) indicated that the straw return method, potassium fertilizer dosage, and ecological region had a significant impact on the maize stem puncture strength, stem compressive strength, and stem bending strength (Table 7). Moreover, there were significant interactions between the straw return method and potassium fertilizer dosage, which affected the above indicators. The interactions between the straw return method and ecological region had a significant influence on the maize stem puncture strength and compressive strength.

**Table 7:** ANOVA results for maize stem lodging resistance mechanical properties under different straw return methods and potassium fertilizer treatments.

Source of variation	DF	Puncture strength	Compressive strength	Bending strength
S	1	141.90***	347.29***	983.80***
K	1	331.64***	792.26***	1609.11***
E	2	12.69***	35.28***	8.48***
S × K	1	40.31***	77.09***	171.30***
S × E	2	5.23**	5.12**	1.81
K × E	2	1.26	2.28	2.55
S × K × E	2	1.18	2.26	1.81

Note: S, K, and E represent straw return treatments, potassium fertilizer treatments, and ecological regions, respectively. Values in the table are F-values with corresponding degrees of freedom for each of the simple effects and two and three way interactions with *, **, *** indicating significant effects at respectively 5%, 1%, 0.1%

**Figure 1:**
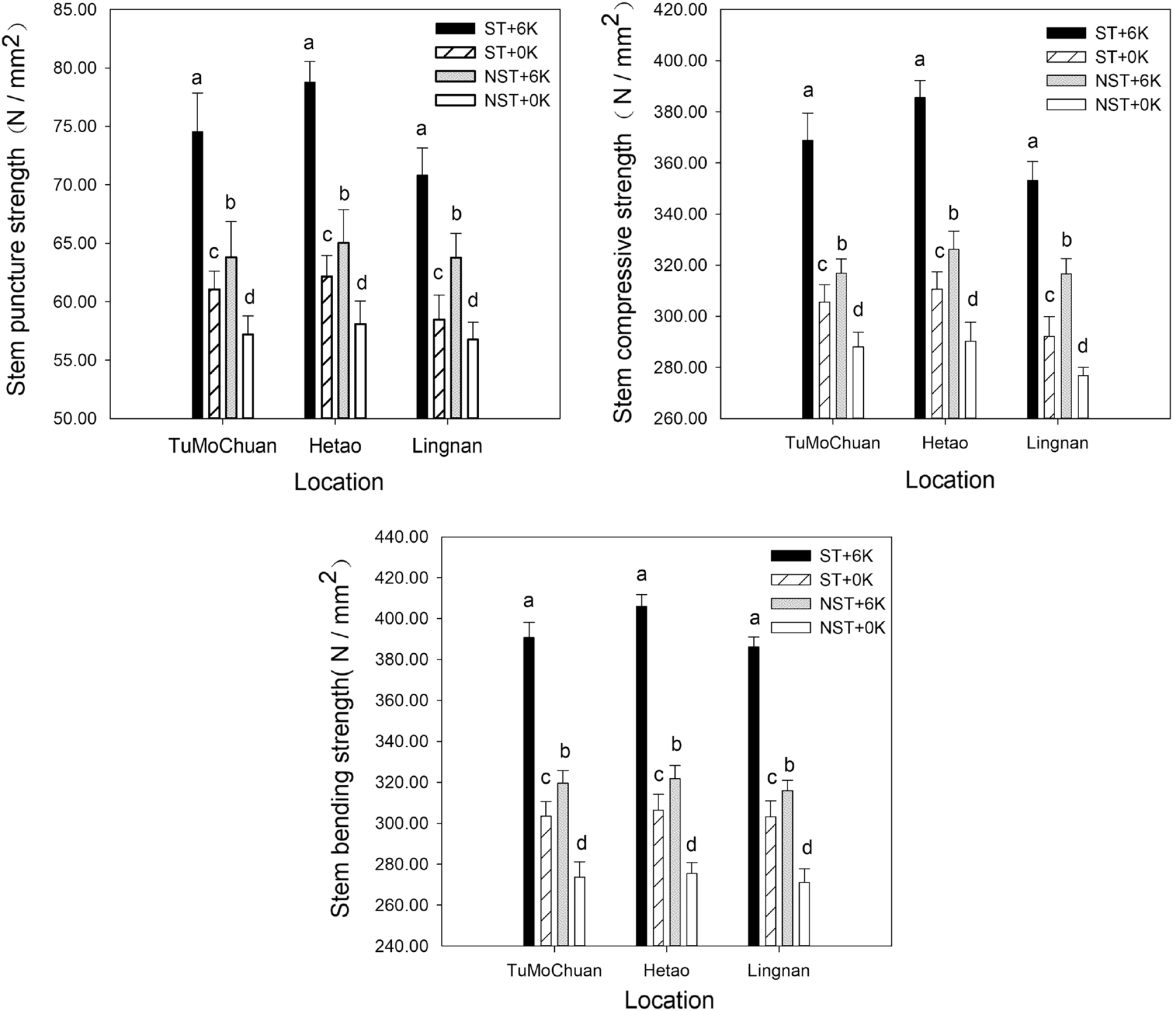
Stem lodging resistance mechanical properties of spring maize under different straw return methods and potassium fertilizer treatments.

The results in Fig. 1 showed that the maize stem puncture strength, stem bending strength, and stem compressive strength varied among the different treatments. In the Tumochuan Plain Irrigation Area, the straw return treatment resulted in significant increases in maize stem puncture strength, stem compressive strength, and stem bending strength. Specifically, under the treatment with straw return, the maize stem puncture strength, stem compressive strength, and stem bending strength increased by 13.49 N/mm^2^, 63.31 N/mm^2^, and 87.41 N/mm^2^, respectively, with potassium application compared to no potassium application. The increase was equivalent to 22.09%, 20.72%, and 28.81%. Under the treatment without straw return, the addition of potassium led to increases of 6.59 N/mm^2^, 28.75 N/mm^2^, and 45.96 N/mm^2^ in the maize stem puncture strength, stem compressive strength, and stem bending strength, respectively, with potassium application compared to no potassium application. These increases represented growth improvements of 11.52%, 9.98%, and 16.79%. It could be concluded that straw return enhanced the effectiveness of potassium application on the maize stem puncture strength, stem compressive strength, and stem bending strength by 10.57%, 10.74%, and 12.01%, respectively.

In the Hetao Plain Irrigation Area, the straw return treatment resulted in significant increases in maize stem puncture strength, stem compressive strength, and stem bending strength. Specifically, under the treatment with straw return, the maize stem puncture strength, stem compressive strength, and stem bending strength increased by 16.61 N/mm^2^, 74.93 N/mm^2^, and 99.65 N/mm^2^, respectively, with potassium application compared to no potassium application. These increases corresponded to a percentage increase of 26.72%, 24.13%, and 32.52%, respectively. Similarly, under the treatment without straw return, the maize stem puncture strength, stem compressive strength, and stem bending strength increased by 6.94 N/mm^2^, 35.89 N/mm^2^, and 46.44 N/mm^2^, respectively, with potassium application compared to no potassium application. These increases represented a percentage increase of 11.94%, 12.36%, and 16.86%, respectively. Straw return enhanced the effectiveness of potassium application on the maize stem puncture strength, stem compressive strength, and stem bending strength by 14.77%, 11.76%, and 15.66%, respectively.

In the Lingnan Warm Dry Zone, the straw return treatment resulted in significant increases in maize stem puncture strength, stem compressive strength, and stem bending strength. Specifically, under the treatment with straw return, the maize stem puncture strength, stem compressive strength, and stem bending strength increased by 12.37 N/mm^2^, 60.83 N/mm^2^, and 83.04 N/mm^2^, respectively, with potassium application compared to no potassium application. These increases corresponded to a percentage increase of 21.15%, 20.82%, and 27.40%, respectively. Similarly, under the treatment without straw return, the maize stem puncture strength, stem compressive strength, and stem bending strength increased by 6.98 N/mm^2^, 39.77 N/mm^2^, and 44.81 N/mm^2^, respectively, with potassium application compared to no potassium application. This represented increases of 12.29%, 14.37%, and 16.53%, respectively. Straw return enhanced the effectiveness of potassium application on the maize stem puncture strength, stem compressive strength, and stem bending strength by 8.86%, 6.45%, and 10.87%, respectively.

### Effects of the straw return method and potassium fertilizer dosage on maize grain quality

The results of the analysis of variance (ANOVA) revealed significant effects of potassium fertilizer dosage and ecological region on the protein content, starch content, crude fat content, and water content of grains (Table 8). Additionally, the straw return method had a significant impact on the protein content, crude fat content, and water content of grains. There were significant interactions between the straw return method and potassium fertilizer dosage, which affected the protein content, crude fat content, and water content of grains.

**Table 8:** ANOVA results for maize grain quality under different straw return methods and potassium fertilizer treatments.

Source of variation	DF	Protein content of grains	Starch content of grains	Crude fat content of grains	Water content of grains
S	1	64.68***	1.66	116.86***	35.96***
K	1	303.95***	7.83***	488.78***	121.18***
E	2	64.35***	11.56***	118.28***	25.62***
S × K	1	16.58***	0.16	9.57***	15.98***
S × E	2	1.97	0.06	2.03	0.21
K × E	2	2.07	0.06	2.28	1.14
S × K × E	2	0.48	0.00	0.34	0.36

Note: S, K, and E represent straw return treatments, potassium fertilizer treatments, and ecological regions, respectively. Values in the table are F-values with corresponding degrees of freedom for each of the simple effects and two and three way interactions with *, **, *** indicating significant effects at respectively 5%, 1%, 0.1%

**Figure 2:**
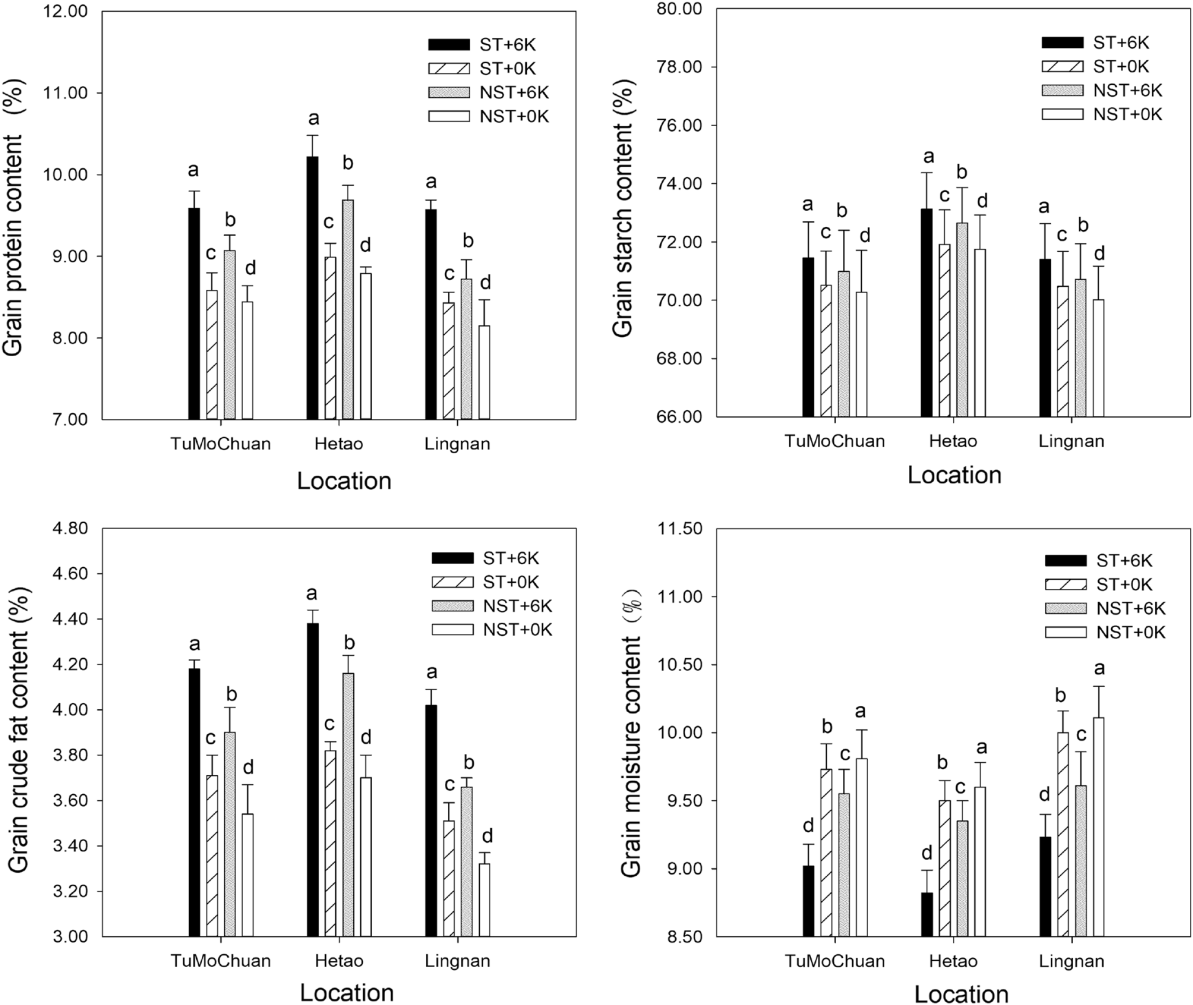
Grain quality of spring maize under different straw return methods and potassium fertilizer treatments.

The protein content, starch content, crude fat content, and water content of maize grains varied significantly among the treatments. Figure 2 illustrated that in the Tumochuan Plain Irrigation Area, Hetao Plain Irrigation Area, and Lingnan Warm Dry Zone, the straw return treatment increased the protein content of grains by 11.77%, 13.68%, and 13.52%, respectively, with potassium application compared to no potassium application, while the starch content of grains increased by 1.33%, 1.68%, and 1.31%. Similarly, the crude fat content of grains increased by 12.67%, 14.66%, and 14.53%, and the water content of grains decreased by 7.30%, 7.16%, and 7.70%, respectively. Under the treatment without straw return, the protein content of grains in the Tumochuan Plain Irrigation Area, Hetao Plain Irrigation Area, and Lingnan Warm Dry Zone increased by 7.46%, 10.24%, and 6.99%, respectively, with potassium application compared to no potassium application. Additionally, the starch content of grains increased by 1.01%, 1.25%, and 1.00%, while the crude fat content of grains increased by 10.17%, 12.43%, and 10.24%, and the water content of grains decreased by 2.65%, 2.60%, and 4.95%. In the Tumochuan Plain Irrigation Area, Hetao Plain Irrigation Area, and Lingnan Warm Dry Zone, straw return increased the effectiveness of potassium application on the protein content of grains by 4.31%, 3.44%, and 6.53%, respectively. Similarly, it enhanced the starch content of grains by 0.32%, 0.43%, and 0.31%, the crude fat content of grains by 2.50%, 2.23%, and 4.29%, and the water content of grains by 4.65%, 4.55%, and 2.75%.

### Effects of straw return method and potassium fertilizer dosage on maize yield

The results of the analysis of variance (ANOVA) indicated significant effects of the straw return method, potassium fertilizer dosage, and ecological region on the maize grain number per ear, 1000-grain weight, and yield (Table 9). Both the straw return method and potassium fertilizer dosage had significant effects on the maize grain number per ear, 1000-grain weight, and yield. Furthermore, the interactions between the straw return method and potassium fertilizer dosage had significant effects on the 1000-grain weight and yield.

**Table 9:** ANOVA results for maize yield and yield component factors under different straw return methods and potassium fertilizer treatments.

Source of variation	DF	Ears per hectare	Grain number per ear	1000-grain weight	Water content	Yield
S	1	0.13	11.23***	38.28***	10.12***	56.97***
K	1	1.03	44.46***	87.38***	25.81***	152.32***
E	2	1.50	8.47***	64.71***	1.75	67.64***
S × K	1	1.04	3.31	7.93**	3.18	12.34***
S × E	2	1.42	0.15	1.82	0.18	2.77
K × E	2	0.16	0.27	0.61	0.18	1.73
S × K × E	2	0.24	0.13	0.54	0.11	1.07

Note: S, K, and E represent straw return treatments, potassium fertilizer treatments, and ecological regions, respectively. Values in the table are F-values with corresponding degrees of freedom for each of the simple effects and two and three way interactions with *, **, *** indicating significant effects at respectively 5%, 1%, 0.1%

As indicated in Table 10, the maize grain number per ear, 1000-grain weight, water content and yield varied among the different treatments. In the Tumochuan Plain Irrigation Area, the straw return treatment resulted in significant increases in maize grain number per ear, 1000-grain weight, and yield. Specifically, under the treatment with straw return, the maize grain number per ear, 1000-grain weight, and yield increased by 31.68 grains/fringe, 45.54 g, and 2476.82 kg/hm^2^, respectively, with potassium application compared to no potassium application. The increase was equivalent to 5.43%, 24.74%, and 21.16%. Under the treatment without straw return, there was an increase of 18.92 grains/fringe, 24.74 g, and 1483.00 kg/hm^2^ in the maize grain number per ear, 1000-grain weight, and yield, respectively, with potassium application compared to no potassium application. These increases represented growth improvements of 3.27%, 8.14%, and 13.17%. It was noteworthy that straw return enhanced the effectiveness of potassium application on the maize grain number per ear, 1000-grain weight, and yield by 2.16%, 6.43%, and 7.99% respectively.

**Table 10:** Maize yield and yield component factors under different straw return methods and potassium fertilizer treatments.

Ecological region	Straw return method	Potassium fertilizer dosage	Spike number (fringes/hm^2^)	Grain number per ear (grains/fringe)	1000-grain weight (g)	Water content (%)	Yield (kg/hm^2^)	Variation coefficient
Tumochuan plain irrigation area	ST	6 K	79,473 ± 814.81a	615.48 ± 15.29a	357.90 ± 14.12a	18.97 ± 0.40d	14,181.86 ± 608.93a	4.29
		0 K	79,902 ± 453.16a	583.80 ± 14.00c	312.36 ± 13.59c	19.66 ± 0.38b	11,705.04 ± 584.26c	4.99
	NST	6 K	80,682 ± 973.64a	597.12 ± 15.07b	328.50 ± 11.41b	19.48 ± 0.37c	12,746.54 ± 665.25b	5.22
		0 K	79,920 ± 1950.49a	578.20 ± 14.52d	303.76 ± 13.01d	19.76 ± 0.39a	11,263.54 ± 645.34d	5.73
Hetao plain irrigation area	ST	6 K	79,842 ± 528.46a	632.20 ± 13.73a	392.46 ± 14.70a	18.75 ± 0.31d	16,091.95 ± 575.74a	3.58
		0 K	79,359 ± 986.05a	593.32 ± 14.46c	342.18 ± 18.76c	19.56 ± 0.41b	12,949.52 ± 554.12c	4.28
	NST	6 K	79,566 ± 671.79a	606.76 ± 14.87b	347.30 ± 11.79b	19.37 ± 0.37c	13,519.84 ± 608.29b	4.50
		0 K	78,924 ± 751.09a	587.16 ± 11.61d	325.28 ± 14.68d	19.74 ± 0.42a	12,096.89 ± 599.79d	4.96
Lingnan warm dry zone	ST	6 K	80,256 ± 672.71a	604.64 ± 15.99a	326.78 ± 12.21a	19.17 ± 0.37d	12,815.56 ± 571.99a	4.46
		0 K	80,313 ± 994.61a	577.36 ± 17.97c	294.12 ± 12.48c	19.74 ± 0.41b	10,945.03 ± 585.23c	5.35
	NST	6 K	79,935 ± 1927.75a	588.84 ± 14.57b	306.24 ± 12.86b	19.54 ± 0.43c	11,598.04 ± 660.44b	5.69
		0 K	79,452 ± 2043.51a	571.48 ± 15.66d	284.02 ± 12.44d	19.89 ± 0.42a	10,333.19 ± 646.47d	6.26

Note: ST represents the straw return treatment. NST represents the non-straw-return treatment. 6 K and 0 K represent the potassium fertilizer dosages.Values are means ± standard deviation; n = 5. Values followed by different letters in a column are significant between treatments using a LSD test (P < 0.05).

In the Hetao Plain Irrigation Area, the straw return treatment resulted in significant increases in maize grain number per ear, 1000-grain weight, and yield. Specifically, under the treatment with straw return, the maize grain number per ear, 1000-grain weight, and yield increased by 38.88 grains/fringe, 50.28 g, and 3142.43 kg/hm^2^ respectively, with potassium application compared to no potassium application. These increases corresponded to a percentage increase of 6.55%, 14.69%, and 24.27%, respectively. Similarly, under the treatment without straw return, the maize grain number per ear, 1000-grain weight, and yield increased by 19.60 grains/fringe, 22.02 g, and 1422.95 kg/hm^2^ respectively, with potassium application compared to no potassium application. These increases represented a percentage increase of 3.34%, 6.77%, and 11.76%, respectively. Straw return enhanced the effectiveness of potassium application on the maize grain number per ear, 1000-grain weight, and yield by 3.21%, 7.92%, and 12.50%, respectively.

In the Lingnan Warm Dry Zone, the straw return treatment resulted in significant increases in maize grain number per ear, 1000-grain weight, and yield. Specifically, under the treatment with straw return, the maize grain number per ear, 1000-grain weight, and yield increased by 27.28 grains/fringe, 32.66 g, and 1870.53 kg/hm^2^, respectively, with potassium application compared to no potassium application. This corresponded to increases of 4.72%, 11.10%, and 17.09%, respectively. Similarly, under the treatment without straw return, the maize grain number per ear, 1000-grain weight, and yield increased by 17.36 grains/fringe, 22.22 g, and 1264.85 kg/hm^2^, respectively, with potassium application compared to no potassium application. These increases represented a percentage increase of 3.04%, 7.82%, and 12.24%, respectively. Straw return enhanced the effectiveness of potassium application on the maize grain number per ear, 1000-grain weight, and yield by 1.68%, 3.28%, and 4.85%, respectively.

Under the straw return treatment, the water content of grains in the Tumochuan Plain Irrigation Area, Hetao Plain Irrigation Area, and Lingnan Warm Dry Zone was reduced by 3.51%, 4.14%, and 2.89%, respectively, with potassium application compared to no potassium application. Similarly, under the treatment without straw return, the water content of grains was reduced by 1.42%, 1.87%, and 1.76%, respectively, with potassium application compared to no potassium application. It was found that straw return increased the effectiveness of potassium application on the water content of grains by 2.09%, 2.27%, and 1.13%, respectively.

In addition, under the straw return treatment, the maize yield variation coefficients in the Tumochuan Plain Irrigation Area, Hetao Plain Irrigation Area, and Lingnan Warm Dry Zone were reduced by 14.03%, 16.36%, and 16.64%, respectively, when potassium was applied compared to no potassium application. Similarly, under the treatment without straw return, the maize yield variation coefficient was reduced by 8.91%, 9.27%, and 9.11%, respectively, with potassium application compared to no potassium application. It was observed that straw return increased the effectiveness of potassium application on the maize yield variation coefficient by 5.13%, 7.08%, and 7.53%, respectively. Furthermore, straw return improved the effectiveness of potassium application on various aspects of maize, including grain number per ear, 1000-grain weight, water content, yield, and yield variation coefficient.

### Effects of straw return method and potassium fertilizer dosage on soil and straw potassium-related indicators after harvest

The results of the analysis of variance (ANOVA) indicated significant influences of the straw return method, potassium fertilizer dosage, and ecological region on the straw potassium accumulation content and soil available potassium content (Table 11). Additionally, the interactions between the straw return method and potassium fertilizer dosage had a significant impact on the straw potassium accumulation content.

**Table 11:** ANOVA results for postharvest soil and straw potassium-related indicators under different straw return methods and potassium fertilizer treatments.

Source of variation	DF	Straw potassium accumulation content	Soil available potassium content
S	1	54.21***	120.45***
K	1	181.76***	40.92***
E	2	76.21***	154.09***
S × K	1	12.74***	1.49
S × E	2	2.52	2.83
K × E	2	1.69	0.32
S × K × E	2	1.11	0.01

Note: S, K, and E represent straw return treatments, potassium fertilizer treatments, and ecological regions, respectively. Values in the table are F-values with corresponding degrees of freedom for each of the simple effects and two and three way interactions with *, **, *** indicating significant effects at respectively 5%, 1%, 0.1%

According to Table 12, the straw potassium accumulation content and soil available potassium content showed variation among the different treatments. In the Tumochuan Plain Irrigation Area, the straw return treatment resulted in significant increases in straw potassium accumulation content and soil available potassium content. Specifically, under the treatment with straw return, the straw potassium accumulation content and soil available potassium content increased by 44.48 kg/hm^2^ and 13.70 mg/kg, with potassium application compared to no potassium application. The increase was equivalent to 27.88% and 12.35%. Under the treatment without straw return, there was an increase of 29.41 kg/hm^2^ and 10.36 mg/kg in the straw potassium accumulation content and soil available potassium content, with potassium application compared to no potassium application. This increase was equivalent to 19.44% and 9.80%. The results indicated that straw return enhances the effectiveness of potassium application, resulting in an 8.44% increase in straw potassium accumulation content and a 2.55% increase in soil available potassium content.

**Table 12:** Postharvest soil and straw potassium-related indicators under different straw return methods and potassium fertilizer treatments.

Ecological region	Straw return method	Potassium fertilizer dosage	Straw potassium accumulation content (kg/hm^2^)	Soil available potassium content (mg/kg)
Tumochuan plain irrigation area	ST	6 K	204.04 ± 7.58a	124.60 ± 5.76a
		0 K	159.56 ± 8.84c	110.90 ± 3.44c
	NST	6 K	180.71 ± 7.82b	116.08 ± 6.95b
		0 K	151.30 ± 7.94d	105.72 ± 4.92d
Hetao plain irrigation area	ST	6 K	242.81 ± 13.53a	149.08 ± 7.00a
		0 K	184.84 ± 9.85c	129.28 ± 3.40c
	NST	6 K	198.79 ± 10.36b	138.04 ± 5.62b
		0 K	171.59 ± 7.78d	121.66 ± 3.77d
Lingnan warm dry zone	ST	6 K	184.95 ± 13.69a	119.08 ± 3.57a
		0 K	148.39 ± 11.20c	106.38 ± 4.42c
	NST	6 K	163.18 ± 12.87b	109.78 ± 4.14b
		0 K	138.98 ± 11.96d	99.58 ± 3.67d

Note: ST represents the straw return treatment. NST represents the non-straw-return treatment. 6 K and 0 K represent the potassium fertilizer dosages. Values are means ± standard deviation; n = 5. Values followed by different letters in a column are significant between treatments using a LSD test (P < 0.05).

In the Hetao Plain Irrigation Area, the straw return treatment resulted in significant increases in straw potassium accumulation content and soil available potassium content. Specifically, under the treatment with straw return, the straw potassium accumulation content and soil available potassium content increased by 57.97 kg/hm^2^ and 19.80 mg/kg, with potassium application compared to no potassium application. The increase was equivalent to 31.36% and 15.31%. Under the treatment without straw return, there was an increase of 27.20 kg/hm^2^ and 16.38 mg/kg in the straw potassium accumulation content and soil available potassium content, with potassium application compared to no potassium application. This increase was equivalent to 15.85% and 13.46%. The results indicated that straw return enhances the effectiveness of potassium application, resulting in an 15.51% increase in straw potassium accumulation content and a 1.85% increase in soil available potassium content.

In the Lingnan Warm Dry Zone, the straw return treatment resulted in significant increases in straw potassium accumulation content and soil available potassium content. Specifically, under the treatment with straw return, the straw potassium accumulation content and soil available potassium content increased by 36.56 kg/hm^2^ and 12.70 mg/kg, with potassium application compared to no potassium application. The increase was equivalent to 24.64% and 11.93%. Under the treatment without straw return, there was an increase of 24.20 kg/hm^2^ and 9.20 mg/kg in the straw potassium accumulation content and soil available potassium content, with potassium application compared to no potassium application. This increase was equivalent to 17.41% and 10.24%. The results indicated that straw return enhances the effectiveness of potassium application, resulting in an 7.23% increase in straw potassium accumulation content and a 1.69% increase in soil available potassium content.

## Discussion

Straw return is an important method for enhancing soil fertility. The decomposition of straw enriches soil nutrients and improves soil fertility. Chen^32^ found that returning straw to the field increased the potassium content in the topsoil and improved soil nutrient availability. In this study, it was found that the accumulation of potassium in straw increased by 44.48 kg/hm^2^ and the available potassium content in the soil increased by 13.70 mg/kg. This can be attributed to the fact that a significant portion of the potassium absorbed by maize was in the form of ions in the straw, accounting for approximately 1.5% of the dry matter mass. After returning straw to the field, this part of the potassium can be quickly released into the soil and absorbed and used by the plants^33^. However, Tan^34^ showed that after 13 consecutive years of straw return, there was no significant increase in soil potassium content in the 0–40 cm soil layer. This can be attributed to the long-term cultivation depleting the soil potassium. Although returning straw to the field can supplement the soil potassium content, it is not sufficient to alleviate soil potassium depletion without the combined application of potassium fertilizer^35^.

Returning straw to the field can improve the plant height and stem diameter of spring maize^36^. Hua^37^ showed that the application of potassium fertilizer can promote the growth of spring maize roots and water absorption, thereby increasing the water content of the stems. Both straw return and potassium fertilizer could enhance the mechanical properties of spring maize stems, significantly improving compressive strength and bending strength and increasing their lodging resistance^38^. In this study, it was observed that straw return further enhanced the effectiveness of potassium fertilizer application on spring maize plant height, ear height, stem fresh weight, stem brix, and stem puncture strength by 2.82–5.22%, 3.11–5.90%, 15.96–19.78%, 4.35–4.50%, and 8.89–14.82%, respectively. This is because returning straw to the field and applying potassium fertilizer increased the potassium supply to spring maize plants, promoted cell division and elongation, and accelerated the stem growth rate^39^. Straw return combined with potassium fertilizer significantly improved the mechanical properties of spring maize stems, with the effectiveness of the treatments ranked as follows: ST+6K > NST+6K > ST+0K > NST+0K. The reason for this is that returning straw to the field and applying potassium fertilizer can improve the structural stability and mechanical strength of spring maize stems, thereby improving the lodging resistance of maize. On the one hand, applying potassium fertilizer can promote the root development and physiological metabolism of maize and improve plant resistance to adversity^22^. On the other hand, returning straw to the field can promote the uptake of potassium by spring maize, which is helpful for increasing stem strength.

Both straw return and potassium fertilizer can improve the protein content, crude fat content and starch content of maize grains, thus improving maize grain quality^40^. Wang^41^ found that straw return is beneficial for improving the protein and amino acid contents of summer maize grains. In this study, straw return combined with potassium fertilizer improved grain quality, with the effectiveness of the treatments ranked as follows: ST+6K > NST+6K > ST+0K > NST+0K. This can be attributed to the fact that potassium is an essential element for plant growth and development and is involved in regulating various physiological processes. Additionally, straw return to the field contributes to improved soil quality. The combined effect of straw return and potassium fertilizer plays a crucial role in enhancing the quality of spring maize grains^42,43^.

Crop yield is a crucial factor in evaluating the impact of fertilizer application on soil productivity^44^. Potassium fertilizer, straw return and the combination of straw return with potassium fertilizer could all significantly increase spring maize yield^45,46^. In this study, it was observed that straw return with potassium fertilizer led to an increase in spring maize yield. The effectiveness of the treatments was ranked as follows: ST+6K > NST+6K > ST+0K > NST+0K. These findings are consistent with previous studies^47,48^. This can be attributed to the conversion of potassium fertilizer into soil available potassium upon entering the soil, which can be directly absorbed and utilized by maize. Compared to potassium contained in potassium fertilizer, potassium in maize straw is more easily fixed by the soil and is not readily released. Straw enters the soil after being returned to the field, and potassium in the returned straw must be released through a long and complex decomposition process through the action of soil microorganisms and enzymes. Therefore, the potassium in the returned straw cannot meet the potassium requirements needed for the growth and development of maize in the current season. Thus, compared to the direct application of potassium fertilizer, short-term straw return has a relatively weaker effect on maize yield.

The variation coefficient of repeated fluctuations in crop yield is an important indicator for evaluating the advantages and disadvantages of different fertilization systems. The main factors that affect the variation coefficient of maize yield are soil fertility and soil basic productivity. When the variation coefficient is relatively small, it indicates higher stability^49^. In this experiment, the optimal model for achieving a stable yield was straw return with potassium fertilizer. The variation coefficients of spring maize yield for the different treatments were ranked as follows: ST+6K < ST+0K < NST+6K < NST+0K. These results are consistent with previous studies. The stable yield achieved in this study can be attributed to the improvement of soil structure and physicochemical properties through straw return with potassium fertilizer. This practice increased soil nutrient content and soil basic productivity, creating a suitable environment for the growth and development of spring maize. As a result, the maize yield variation coefficient was reduced, leading to increased yield stability^50,51^.

The results of the study indicated that the practice of returning straw to the field and applying potassium fertilizer could enhance the lodging resistance, grain quality, and yield of spring maize. However, there is a need to further explore and address the specific physiological mechanisms behind this growth promotion in maize under application of straw and potassium fertilizer. Future research can further elucidate the mechanism of straw return and potassium fertilizer application by analysing indicators such as soil enzyme activity, microbial community structure, and changes in soil fertility to gain a more comprehensive understanding of the effects on maize growth. Second, the study showed that the spring maize stem morphological indicators, stem phenotypic traits, stem mechanical properties, grain quality, and yield were different in the three tested ecological regions in Inner Mongolia, yet the trends in the changes in relevant measurements in each of these areas due to the application of straw return with potassium fertilizer were extremely similar. Among the tested ecological regions, each of these factors was superior in the Hetao Plain irrigation area, followed by the Tumochuan Plain irrigation area, and finally the Lingnan Warm dry zone. These differences were mainly due to differences in factors such as basic soil productivity and climatic conditions (sunshine hours, temperature, and rainfall) among the regions. Future work can further explore the effects of straw return and potassium fertilizer combination application under different conditions to provide more specific guidance and suggestions.

## Conclusions

This study investigated the effects of straw return methods and potassium fertilizer application rates on the morphological indexes, phenotypic traits, stem lodging resistance mechanical properties, grain quality, and yield of spring maize in three ecological regions from east to west Inner Mongolia. The study examined suitable cultivation models for high-quality and high-yield of maize. The study provides a basis for the development of the green crop industry and the stress resistance of maize. The research results indicate that straw return increases the effectiveness of potassium fertilizer application on spring maize plant height, ear height, stem diameter, stem dry weight, stem puncture strength, grain protein content, grain starch content, grain crude fat content, maize yield, yield variation coefficient, straw potassium accumulation content, and soil available potassium content by 2.82–5.22%, 3.11–5.90%, 4.31–6.04%, 8.91–11.68%, 8.86–14.77%, 3.44–6.53%, 0.31–0.43%, 2.23–4.29%, 4.85–12.50%, 5.13–7.53%, 7.23–15.51%, and 1.69–2.55%, respectively. Both straw return and potassium application can improve stem lodging resistance, grain quality, and maize yield. Straw return can be combined with the reasonable application of potassium fertilizer to increase the effectiveness of potassium fertilizer, enhance lodging resistance, increase maize yield, and improve grain quality and yield stability.

This study provided relevant information for agricultural fields, deepened the effects of the interaction between straw return and potassium fertilization on maize traits and yield, and provided beneficial guidance for the sustainable development of agricultural production. Further research can examine the effectiveness of soil improvement measures to improve straw return and potassium fertilizer application in different soil types and qualities. This study can help in efficient utilization of resources to promote sustainable agricultural development.

## Data Availability

The datasets used and/or analysed during the current study are available from the corresponding author upon reasonable request.
